# Shifts in rumen prokaryotic communities of grazing beef cows with divergent feed efficiency in response to seasonal grazing

**DOI:** 10.1186/s42523-026-00581-6

**Published:** 2026-11-10

**Authors:** Sang Weon Na, Mi Zhou, Youyoung Choi, Hanbeen Kim, Yanhong Chen, Edward W. Bork, Carolyn Fitzsimmons, Le Luo Guan

**Affiliations:** 1 Department of Agricultural, Food & Nutritional Science, University of Alberta, Edmonton, AB T6G 2P5 Canada; 2 Faculty of Land and Food Systems, The University of British Columbia, Vancouver, BC V6T 1Z4 Canada; 3 Lacombe Research and Development Centre, Agriculture and Agri-Food Canada, Lacombe, AB Canada

**Keywords:** Cow-calf production, Feed efficiency, Grazing, Metataxonomic sequencing, Rangeland, Rumen microbiome

## Abstract

**Background:**

Cow–calf operations represent a major component of beef production, typically relying on seasonal grazing of perennial forages. While understanding the role of the rumen microbiome in improving feed efficiency in grazing systems is essential for sustainable beef production, most studies have focused on cattle in confined systems such as feedlots. There is limited understanding of how rumen microbial communities change across feeding regimens in cow-calf operations with seasonal grazing. This study characterized rumen prokaryotic communities of beef cows across four feeding regimens, drylot (DL), planted pasture (PP), summer native rangeland (SNR), and fall native rangeland (FNR) within a year-round cow–calf production cycle, aiming to determine their relationships with feed efficiency.

**Results:**

Prokaryotic communities in rumen samples collected from breeding beef cows under DL, PP, SNR, and FNR were assessed using metataxonomic sequencing and quantitative PCR. Feed efficiency was evaluated using residual feed intake adjusted for backfat thickness (RFIf) under DL. The microbial profiling and quantification revealed significant shifts in microbial composition and diversity across the four regimens. Bacterial diversity was highest in DL and declined during grazing, while archaeal diversity increased in SNR compared to PP and FNR. Several taxa were differentially enriched under distinct feeding regimens: *Thermobifida*, *Saccharomonospora*, *Corynebacterium*, and *Methanoculleus thermophilus* appeared only in DL, while Patescibacteria and *Olsenella* were more abundant during grazing. Further analysis of microbial fermentation profiles revealed that concentrations of major volatile fatty acids (VFAs) were higher in PP than in the other regimens. Heritable bacteria (*p*-251-o5 and *Pirellula*) were enriched in native rangelands and negatively correlated with VFA concentrations. In FNR, the abundance of the dominant bacterial taxon *Christensenellaceae* R-7 group increased, while *Prevotella* declined. Differential abundance analysis identified RFIf-associated taxa, including *Eubacterium cellulosolvens* group, *Colidextrobacter*, and *Methanosphaera* sp. ISO3–F5 regardless of feeding regime variation.

**Conclusions:**

This study provides a comprehensive assessment of rumen prokaryotic dynamics across seasonal feeding regimens in cow-calf production, revealing varied shifts between bacteria and archaea. We further identified feeding regimen-specific associations between microbial taxa and feed efficiency, suggesting diet-microbiome interactions influencing grazing cow’s feed efficiency. These findings enhance the understanding of host-microbe interactions in confined and grazing environments, offering insights for sustainable beef production strategies.

**Supplementary information:**

The online version contains supplementary material available at https://doi.org/10.1186/s42523-026-00581-6.

## Background

The cow-calf phase is a major component of the Canadian beef cattle industry, commonly utilizing open-range grazing during spring, summer and fall in regions with abundant perennial forage lands [1]. Generally, cattle graze on cultivated (i.e., planted) pastures or native rangelands during the growing season and are fed stored hay or silage during cold seasons. In Canada, cow-calf operations account for 66% of the total beef cattle herd, most of which (80%) are located in the Canadian Prairies, encompassing vast areas of native (never cultivated) rangeland (13 M ha) and planted pasture (5 M ha) [2–4]. By utilizing grazing lands, cow-calf operations benefit from reduced feed costs and improved animal welfare, and are compatible with native rangeland conservation that in turn supports carbon sequestration [5] and enhances ecosystem biodiversity [6]. However, overgrazing can generate negative environmental impacts such as soil erosion and water pollution [7, 8]. Furthermore, compared to feedlot cattle, grazing cattle produce higher levels of enteric methane, a major greenhouse gas that accelerates climate change [9, 10], accounting for 80% of total enteric methane emissions across the entire beef cycle [11]. The cow-calf phase presents a significant challenge for ensuring the beef cattle industry’s sustainability, particularly in minimizing its negative environmental footprint.

Improving feed efficiency (FE) has been reported to be associated with reduced methane emission in cattle [12–14]. FE, a key economic trait in beef cattle production, influences both profitability (i.e., feed costs) and environmental impact. Metrics such as feed conversion ratio (FCR), residual average daily gain (RADG), and residual feed intake (RFI) are commonly used to evaluate the FE of cattle [15]. Among them, RFI has gained attention for its moderate heritability and independence from body weight or size [16]. Most RFI-based feed efficiency studies have been conducted under confined conditions (feedlot or drylot), while it is difficult to measure the feed intake of cattle while grazing [16–18]. It is now widely accepted that the rumen microbiome can directly affect the cattle FE due to its essential role in digesting complex plant carbohydrates and providing important nutrients to the host [19]. The rumen microbiome consists of a diverse and complex microbial community, including bacteria, archaea, protozoa, fungi, and bacteriophages [20]. These microbes degrade and ferment ingested feed to produce microbial products such as volatile fatty acids (VFAs), microbial proteins, lipids, and vitamins, which serve as the primary energy and nutrient sources for the host [21]. Given the importance of rumen microbes in digestion and nutrition, numerous studies have reported their functions in relation to cattle FE [22–28]. The linkage between rumen bacterial community profiles and their fermentation traits, and their relationship with feed efficiency was first assessed by Guan et al. [22], and the latter studies by Carberry et al. [24] and Elolimy et al. [29] reported lower abundances of *Prevotella* and *Succinimonas amylolytica* in efficient beef cattle compared to inefficient cattle, respectively. Additionally, Li et al. [30, 31] reported that inefficient beef cattle had a more diverse microbial composition and functions than efficient beef cattle. However, most of these studies have been conducted under feedlot conditions, with limited research examining changes in the rumen microbiome of beef cows with different FE across different feeding regimens, including seasonal grazing. We hypothesized that seasonal transitions between drylot feeding and grazing would drive systematic shifts in rumen prokaryotic communities and fermentation profiles, which would be associated with RFIf.

Therefore, in the present study, we investigated the composition and diversity of rumen bacterial and archaeal communities in beef cows across four different feeding regimens (feeding in drylot, planted pasture, summer native rangeland, and fall native rangeland) within an annual cow-calf production cycle. In addition, we explored the relationship between changes in the rumen prokaryotic communities and RFIf, which was evaluated when they were yearlings in drylot.

## Materials and methods

### Animals, grazing seasons, and sampling

Kinsella Composite crossbred cows (*n* = 37; average body weight = 545.3 ± 39.6 kg), born in 2018 and raised at the Roy Berg Kinsella Research Ranch (53°0.27′N, 111°31.2′W), University of Alberta (AB, Canada), were used for this study. The Kinsella Composite crossbred was developed by crossing beef lines (Angus, Charolais, Galloway, or Hereford) and dairy lines (Brown Swiss, Holstein, and Simmental) as described in Olson et al. [32].

**Fig. 1:**
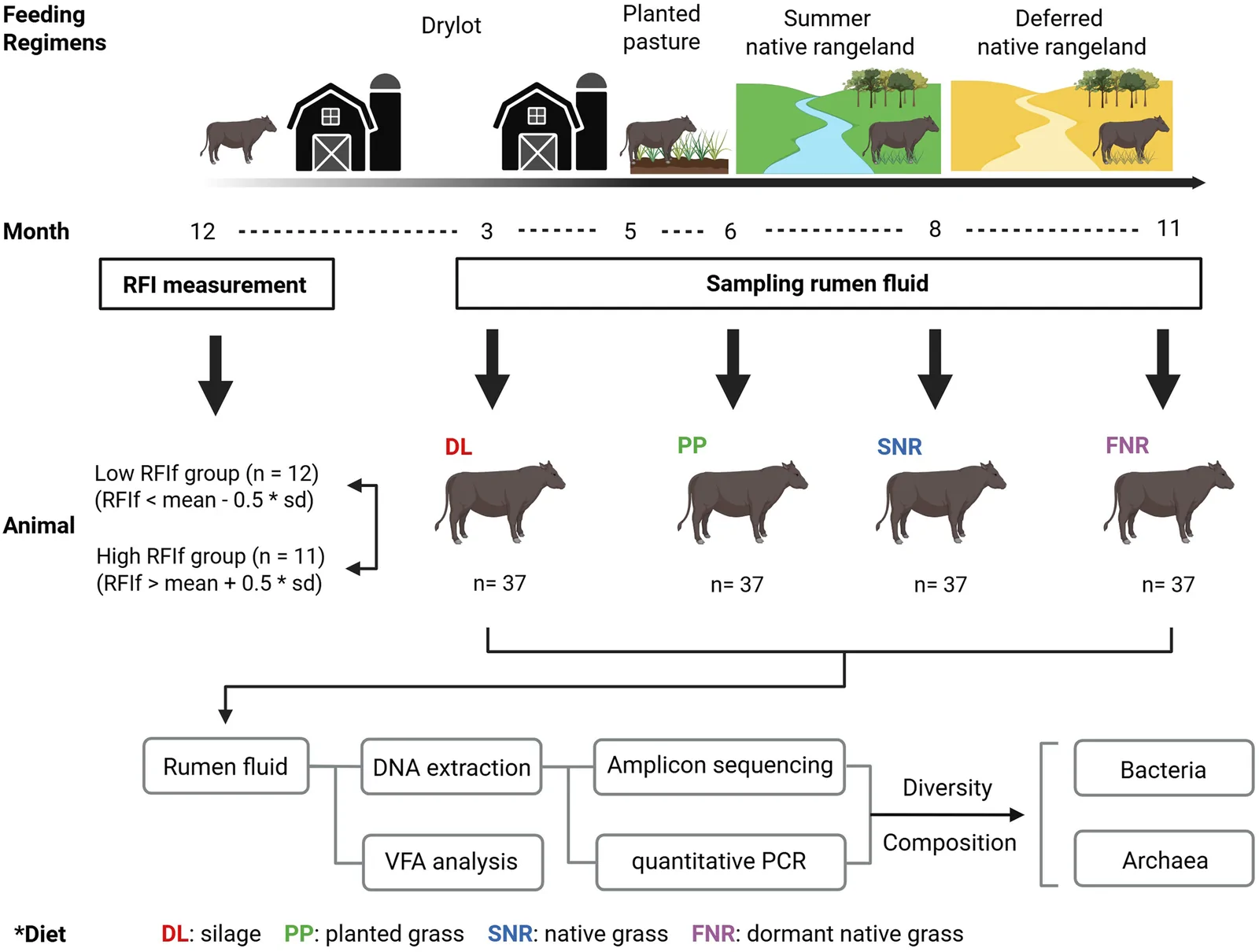
Overview of experimental design

The experimental design is illustrated in Fig. 1. Cows were examined across four different feeding regimens of the annual cow-calf production cycle, beginning in December 2020 and ending in November 2021. These regimens included: 1) confined feeding under drylot (DL; December to April), 2) spring grazing on planted pasture (PP; May to June), 3) open-range grazing of native rangeland in summer during peak growth (SNR; July to August), and 4) open-range grazing of native rangeland during the dormant season (FNR; September to November). Under DL during winter, the cows were fed a silage mixture of 60% barley and 40% oats with a nutrient analysis on a dry matter (DM) basis showing 13.3% crude protein, 33.1% acid detergent fibre, 48.5% neutral detergent fibre, 0.4% calcium, 0.4% phosphorus, 0.2% magnesium, 2.2% potassium, 0.2% sodium, and 60.8% total digestible nutrients, and trace minerals including 336 ppm iron, 65.3 ppm manganese, 31.3 ppm zinc, and 5.7 ppm copper. Dominant plants in the spring (planted) pasture were *Bromus inermis* Leyss (Smooth brome), *Poa pratensis* L. (Kentucky bluegrass), *Phleum pratense* (Timothy), *Elymus repens* (quackgrass), and *Medicago sativa* L. (Alfalfa). Grazing during summer and fall took place in the native rangelands comprising a diverse mix of vegetation types, including upland grassland, shrubland, forest and wetlands. Common plant species in the landscape included *Populus tremuloides* (Trembling aspen), *Bromus inermis* Leyss (Smooth brome), *Poa pratensis* L. (Kentucky bluegrass), *Elymus trachycaulus* (Link) Gould (Slender wheatgrass), *Carex* species (Sedge – upland spp.), *Symphoricarpos occidentalis* Hook. (Western snowberry), *Elaeagnus commutata* (Wolf willow), *Amelanchier alnifolia* (Nutt.) (Saskatoon), and *Distichlis spicata* (L.) Greene (Saltgrass). The cattle gave birth to calves between April 19 and May 26, 2021, and were actively nursing their calves during all grazing periods. At the end of each regimen of the cow-calf production cycle (March, June, August and November), cattle were handled and approximately 50 mL of rumen sample were collected from between 09:00 and 14:00 using an oro-gastric tube. Samples were immediately frozen in dry ice and subsequently stored at −80 °C until DNA extraction and VFA analysis.

### Feed efficiency measurement

RFI was assessed over three months under DL from December 2020 to February 2021, but not during grazing periods due to the facility limitation. The GrowSafe feed intake monitoring system (Vytelle Technology Centre, Calgary, AB, Canada) was used to collect individual daily intake of feed, while body weight was recorded biweekly to determine average daily gain (ADG). Dry matter intake (DMI) was calculated from the daily feed intake of the cows and the DM proportion (%) of the feed. Standardized DMI was calculated by dividing each animal’s total metabolizable energy (ME) intake by 10, assuming a uniform energy density of 10 MJ ME/kg DM. The resulting DMI was then divided by the number of test days to calculate the average standardized daily DMI (SDMI, kg DM/day). RFI was calculated and adjusted following the method of Basarab et al. [33], by regressing SDMI (Y_ijk_) on ADG, metabolic body weight (MBW), and final ultrasound backfat thickness (FUFAT). The expected feed intake (EFI = β_0_ + β_1_ × ADG_i_ + β_2_ × MBW_j_ + β_3_ × FUFAT_k_) for each animal was obtained from the regression equation, and the adjusted RFI by ultrasound backfat thickness (RFIf) was calculated as the difference between EFI and actual SDMI.

Regression model: Y_ijk_ = β_0_ + β_1_ × ADG_i_ + β_2_ × MBW_j_ + β_3_ × FUFAT_k_ + e_ijk_

The animals with the top 12 and bottom 12 RFI values were then selected to collect rumen fluid for microbial profiling.

### DNA extraction

Total DNA was extracted from a 3 mL aliquot of rumen fluid using a repeated bead-beating plus column method as described by Guan et al. [22]. Briefly, 1.5 mL of rumen fluid was transferred to a tube containing 0.5 g of 0.1 mm silica beads and centrifuged at 19,000 × *g* for 5 min at 4 °C, and the supernatant was discarded. An additional 1.5 mL of rumen fluid was added to the tube and centrifugation repeated to remove the supernatant. Pellets were washed with 1 mL Tris-EDTA buffer (10 mM Tris-HCl and 1 mM EDTA) by vortexing and centrifugation at 19,000 × *g* for 5 min at 4 °C. After removing the supernatant, cells were lysed with lysis buffer and homogenized by physical disruption using bead beating with a BioSpec Mini Bead-Beater (BioSpec, Bartlesville, OK, USA) at 4,800 rpm for 3 min. The supernatant was then processed following the protocol supplied with the QIAamp fast DNA mini-Stool kit (Qiagen, Hilden, Germany) for removal of protein and DNA purification. DNA quality and quantity were measured at A260/A280 using an ND-1000 spectrophotometer (Thermo Fisher Scientific, Wilmington, DE, USA).

### Assessment of microbial abundance using quantitative PCR analysis

Total populations of rumen bacteria and archaea were estimated by calculating the copy number of the marker genes using quantitative PCR (qPCR) with universal primers: total bacteria (U2-F: 5’-ACTCCTACGGGAGGCAG-3’; U2-R: 5’-GACTACCAGGGTATCTAATCC-3’) [34] and total archaea (uniMet1-F: 5’-CCGGAGATGGAACCTGAGAC-3’; uniMet1-R: 5’-CGGTCTTGCCCAGCTCTTATTC-3’) [23], respectively. Standard curves were constructed using serial dilutions of purified plasmid containing 16S rRNA genes of *Butyrivibrio hungatei* for total bacteria and *Methanobrevibacter* sp. strain AbM4 for total archaea to establish the relationship between PCR results and gene copy numbers, allowing accurate quantification of microbial populations in the rumen samples [23, 35]. SYBR Green chemistry (Fast SYBR Green Master Mix) was used for qPCR in the StepOnePlus Real-time PCR System (Applied Biosystems, Foster City, CA, USA). The PCR programs were as follows: for bacteria, the holding stage started at 95 °C for 5 min and was followed by 40 amplification cycles (95 °C for 20 s, 60 °C for 1 min); for archaea, the starting temperature was 95 °C for 20 s, followed by 40 amplification cycles (95 °C for 3 s and 60 °C for 30 s), with fluorescent signals collected at each cycle. A melting curve analysis was performed after amplification, starting at 95 °C for 15 s, followed by 60 °C for 1 min. The temperature was then increased by 0.3 °C every 20 s from 60 °C to 95 °C and held at 95 °C for 15 s, to assess the specificity of the PCR products. The equation to calculate the abundance of each microbial group, as described by Li et al. [35], is shown below:

DNA gene copy number = (6.02 × 10^23^ (molecules/mol) ×DNA amount (g))/(DNA length (bp) ×660(g/mol/bp))

### Ruminal volatile fatty acid (VFA) profiling

The concentration of VFAs was measured by gas chromatography (GC). Rumen fluid (4 mL) was transferred to a 15 mL tube and centrifuged at 13,000 rpm for 5 min at 4 °C. The supernatant was thoroughly mixed with 25% phosphoric acid at a 4:1 (v/v) ratio of supernatant to acid and 1 mL of the mixture was mixed with 0.2 mL of an internal standard solution. After overnight incubation at −20 °C, samples were centrifuged at 19,000 × *g* for 5 min at 4 °C and then transferred to a 1.8 mL GC vial. The concentrations of acetate, propionate, butyrate, iso-butyrate, valerate, and iso-valerate were determined using a Varian 430-GC (Varian, Walnut Creek, CA, USA) with a Stabilax-DA column (Restek Corp., Bellefonte, PA, USA).

### Amplicon sequencing and bioinformatics analysis

Bacterial and archaeal partial 16S rRNA genes were amplified using primer sets targeting the bacterial V1–V3 region (FAM27f: GAGTTTGATCMTGGCTCAG; Bac515R: CCGCGGCKGCTGGCAC) [36, 37] and the archaeal V6–V8 region (AR934F: AGGAATTGGCGGGGGAGCAC; Arc1386R: GCGGTGTGTGCAAGGAGC) [38, 39]. The amplicon sequencing libraries were constructed following the 16S Metagenomic Sequencing Library Preparation Protocol from Illumina and sequenced using an Illumina MiSeq instrument (300 bp × 2 paired-end; Illumina, San Diego, CA, USA) at the Agricultural Genomics and Proteomics unit (University of Alberta, AB, Canada). Of the 24 samples sequenced, one sample failed to generate proper data for downstream analysis, and the sequence data from the remaining 23 samples were used for microbial taxonomy and functional analysis (detailed RFI of the samples were listed in Table S1).

Raw sequencing reads were trimmed and filtered based on the read quality (Phred score of 25). The filtered reads were then inferred as amplicon sequence variants (ASVs) with default cutoffs for error modeling (OMEGA_A = 1e-40, OMEGA_C = 1e-40, and band size = 16), and paired reads were merged by DADA2 (v. 3.11) [40]. ASV tables were used to calculate alpha and beta diversity, and for taxonomic annotation by QIIME2 (v. 2024.5) [41]. Alpha and beta diversity of the rumen bacterial and archaeal communities were calculated using ASV tables rarefied to the lowest read count among samples (Bacteria: 3,000; Archaea: 2,100), meaning that each sample was subsampled to an equal sequencing depth. The alpha diversity indices included observed ASVs, observed genera, Chao1, Shannon, Simpson, Pielou’s evenness, and Faith’s phylogenetic diversity (Faith’s PD). The beta diversity was assessed based on the weighted UniFrac distance matrices between samples, and ordination was performed using principal coordinate analysis using the ape (v. 5.8) package in R. SILVA small subunit rRNA database (v. 138.1) [42] and RIM-DB [43] were used for taxonomic annotation of bacteria and archaea. For further analyses including taxonomic profiling, differential abundance analysis (DAA), and correlation analysis, we selected only taxa that had a relative abundance greater than 0.1% in at least 12.5% of all samples. The remaining taxa were combined into a single category labeled “Others”.

### Predicted functional analysis using CowPI and PICRUSt2-SC

CowPI [44] was employed to investigate the functional potential of rumen microbes based on KEGG Level 3 pathways, focusing on major functional features. Additionally, PICRUSt2-SC (v2.6.1, released March 04, 2025) [45] was used to predict the potential functions of bacteria and archaea based on KEGG Orthology, modules, and pathways. Following the developers’ recommendations, bacterial and archaeal sequences were placed into their respective reference phylogenies. The results from both analyses were then combined for downstream analysis.

### Identification of microbial taxa affected by feeding regimens and feed efficiency

Significantly different taxa at the phylum and genus levels were identified between feeding regimens and RFIf groups using microbiome multivariate association with linear models (MaAslin2) (v. 1.16.0) [46] package in R. The same analytical framework was also applied to the predicted functional profiles (KO, KEGG pathways, and KEGG modules) derived from CowPI and PICRUSt2-SC. For DAA using MaAslin2, the different feeding regimens of the cow-calf production cycle and RFIf factors were included as fixed effects, and individual animal was considered a random effect for the linear mixed model. The model coefficient values (coef; log2-transformed effect sizes, e.g., coef: 1.5 = ~2.83 × higher abundance) and significance (*P-*value) of the associations between microbial taxa and fixed effects were calculated. The false discovery rate (FDR) of the *p*-value was corrected using the Benjamini-Hochberg method with an FDR threshold of 0.10 for DAA, and the adjusted *P-*values were defined as *Q*-values. The strength of differential abundance was classified based on the absolute values of their model coefficients: weakly (|coef| <2), moderately (2 ≤ |coef| <4), and strongly (|coef| ≥4). DAA included all pairwise comparisons among feeding regimens; however, we focused on comparisons between consecutive feeding stages (DL vs. PP, PP vs. SNR, and SNR vs. FNR), as adjacent time points allowed for clearer biological interpretation and minimized variability across long time intervals.

### Statistical analysis

Post hoc power analysis based on the observed RFI values using G*Power 3.1.9.7 [47] showed that the sample size of the current study (*n* = 12 for L-RFI and 11 for H-RFI group) achieved > 0.99 power at α = 0.05 to detect differences between RFI groups for all analyses. The normality of all data distributions was assessed using the Shapiro-Wilk test using built-in stats package in R (v. 4.3.2).

Concentrations of VFAs, microbial populations, and alpha diversities among the feeding regimens in the cow-calf production cycle were compared using non-parametric methods. First, we conducted the Friedman test using rstatix (v. 0.7.2) in R to assess overall differences across the repeated measures collected from the same animals under four different feeding regimens (DL, PP, SNR, and FNR). Then we performed the Nemenyi test using the PMCMRplus (v. 1.9.12) package in R for multiple comparisons between feeding regimens. Differences between RFIf groups were evaluated with the Wilcoxon rank-sum test using rstatix (v. 0.7.2) in R for pairwise comparisons. The interaction between the feeding regimens and RFIf was assessed by comparing generalized linear mixed models with and without the interaction term using ANOVA with the lme4 (v. 1.1–35.5) and built-in stats packages in R. Permutational multivariate analysis of variance (PERMANOVA) and the pairwise comparisons of the feeding regimens were performed with 999 permutations using the vegan (v. 2.6–8) package in R to analyze microbial community beta-diversity based on the weighted UniFrac distance matrices for bacteria and archaea. To identify the taxa contributing most to the dissimilarity between groups, similarity percentage (SIMPER) analysis was performed using Bray–Curtis dissimilarity on relative abundance data with 999 permutations in the vegan package in R to identify taxa contributing to group dissimilarity, conducted at the ASV level for bacteria and at the species level for archaea.

To investigate the relationship between VFAs and rumen prokaryotic alpha diversity and relative abundance, a Spearman’s rank correlation analysis was performed using the Hmisc (v. 5.2–0) package in R. The strength of correlation was interpreted based on the absolute value of the correlation coefficient (ρ): weak (|ρ| < 0.3), moderate (0.3 ≤ |ρ| < 0.7), and strong (|ρ| ≥ 0.7). *P*-values were calculated by asymptotic t-distribution approximation and adjusted using the Benjamini-Hochberg procedure at the FDR threshold of 0.05. *P-*values and *Q*-values less than 0.05 were considered statistically significant, while those between 0.05 and 0.1 were interpreted as indicating a trend.

## Results

### Changes in total microbial populations and VFA profiles across feeding regimens and RFIf groups

**Fig. 2:**
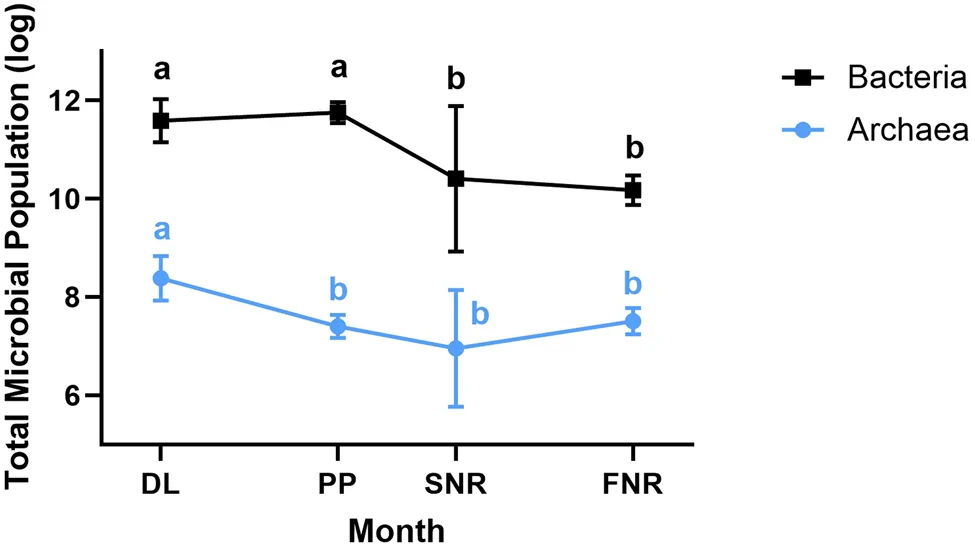
Differences in rumen bacterial and archaeal populations across the feeding regimens of the cow-calf production cycle quantified by qPCR. DL: confined feeding in drylot; PP: spring grazing in planted pasture; SNR: summer grazing in native rangeland; FNR: fall grazing in dormant native rangeland. Different letters indicate significant differences (*p* < 0.05) among the feeding regimens based on the Nemenyi post-hoc test

Analysis with qPCR revealed that total bacterial and archaeal populations estimated by their respective 16S rRNA gene copy numbers ranged from 10^9^ to 10^12^ gene copy numbers/mL of rumen fluid and from 10^6^ to 10^9^ gene copy numbers/mL for bacteria and archaea, respectively. Both bacterial and archaeal populations were significantly affected by the feeding regimen (*p* < 0.001 for overall effect) (Fig. 2). Pairwise comparisons between feeding regimens showed that total bacterial populations were similar in the rumen of cows fed in DL and PP but significantly decreased when cattle grazed in SNR and FNR (*p* < 0.001). In contrast, the total methanogenic population decreased in the rumen of cows from DL to those grazing in PP, SNR, and FNR (*p* < 0.001).

Concentrations of total VFA and major VFAs, including acetate, propionate, and butyrate, were significantly higher in the rumens of cattle grazing in PP compared to all other feeding regimens (DL, SNR, and FNR) (Table 1; *p* < 0.001). Meanwhile, concentrations of iso-butyrate and iso-valerate in the FNR were significantly lower than in other feeding regimens (*p* < 0.05) (Table 1). The proportions of acetate and acetate-to-propionate ratio were the lowest in the rumen of cows in PP among the feeding regimens (*p* < 0.01), while the proportions of propionate and butyrate were higher in PP than in DL (*p* < 0.001). Iso-butyrate and iso-valerate were present in higher proportions in DL than in PP and FNR (*p* < 0.001), but proportionally lowest in FNR compared to all prior feeding regimens. Total prokaryotic populations and VFAs did not differ between the RFIf groups (*p* > 0.10).

**Table 1:** Profiles of rumen volatile fatty acids in beef cows across the feeding regimens of the cow-calf production cycle

Items	**Treatment**^**1**^				**SEM**^**2**^	*P***-values**^**3**^
	DL	PP	SNR	FNR		
Concentration (mM)						
Total VFA	28.3b	42.3a	27.1b	26.3b	1.15	<0.001
Acetate	20.3b	29.5a	19.3b	19.3b	0.79	<0.001
Propionate	4.54b	7.16a	4.23b	4.40b	0.20	<0.001
Butyrate	2.48b	4.47a	2.66b	2.20b	0.14	<0.001
Iso-butyrate	0.35a	0.37a	0.29a	0.14b	0.01	<0.001
Valerate	0.19b	0.33a	0.26ab	0.13c	0.01	<0.001
Iso-valerate	0.50a	0.49ab	0.36b	0.11c	0.02	<0.001
Proportion (%)						
Acetate	71.7b	69.8c	71.3b	73.5a	0.18	<0.001
Propionate	16.0b	16.9a	15.6b	16.8a	0.10	<0.001
Butyrate	8.66b	10.5a	9.69a	8.27b	0.13	<0.001
Iso-butyrate	1.24a	0.87b	1.10a	0.54c	0.03	<0.001
Valerate	0.68b	0.78ab	0.94a	0.49c	0.02	<0.001
Iso-valerate	1.76a	1.17b	1.34b	0.43c	0.05	<0.001
Acetate:propionate ratio	4.50a	4.14b	4.58a	4.38a	0.03	<0.001

^1^Cows were fed across four different feeding regimens of the annual cow-calf production cycle: (1) confined feeding in drylot (DL); spring grazing in planted pasture (PP); summer grazing in native rangeland (SNR); and fall grazing in dormant native rangeland (FNR)

^2^SEM, standard error of the mean

^3^*P*-values were obtained from the Friedman test, which assess overall differences across all the feeding regimens

^a,b,c^ Means within a row with different superscript letters differ significantly based on the Nemenyi post-hoc test (*p* < 0.05)

### Temporal dynamics of rumen microbial diversities across feeding regimens and RFIf groups

A total of 31,087 ± 6,423 and 13,286 ± 2,726 (mean ± standard deviation) raw reads were obtained from each rumen sample for bacteria and archaea, respectively. After filtering and denoising, an average of 5,717 ± 1,662 and 8,801 ± 2,062 (mean ± standard deviation) reads were obtained from the bacterial and archaeal communities, respectively (Tables S2 and S3). A comparison of alpha diversity of rumen prokaryotic communities across the feeding regimens and between RFIf groups revealed that the alpha diversity indices (observed ASVs, observed genera, Chao1, Shannon and Simpson diversity, and Faith’s PD) of all prokaryotes were significantly affected by the feeding regimens (*p* < 0.01), except for Pielou’s evenness in the archaeal communities (Table 2). All the bacterial alpha diversity indices of rumen microbiota in DL, except for Pielou’s evenness, were significantly higher than those under the subsequent periods when cattle were grazing (PP, SNR, and FNR) (Table 2; *p* < 0.05). In archaeal communities, the SNR group showed higher values in Chao1, Shannon, and Faith’s PD compared to the other grazing regimens (PP and FNR) (Table 2; *p* < 0.05). Bacterial alpha diversities were not significantly affected by RFIf, but archaeal communities had significantly higher Shannon (L-RFIf: 4.52 ± 0.22, vs H-RFIf: 4.35 ± 0.27) and Simpson diversities, as well as Pielou’s evenness, in the L-RFIf group compared to cows from the H-RFIf group (Table 2; *p* < 0.05). The interaction effects between feeding regimens and RFIf on bacterial and archaeal communities were not detected (Table 2).

**Table 2:** Alpha diversity metrics of rumen bacterial and archaeal communities in high and low RFIf cow groups across the feeding regimens

**Feeding regimen**^**1**^	DL		PP		SNR		FNR		*P-***values**^**3**^		
**RFIf**^**2**^	H	L	H	L	H	L	H	L	Feeding regimen	RFIf	Interaction
Bacteria											
Observed ASVs	737 ± 130	708 ± 120	620 ± 75.8	624 ± 124	643 ± 115	625 ± 135	553 ± 91.8	548 ± 47.7	<0.001	0.40	0.95
Observed genera	91.5 ± 16.3	86.2 ± 8.62	77.6 ± 6.79	75.9 ± 10.6	74.4 ± 8.18	71.3 ± 11.6	71.2 ± 4.29	69.1 ± 3.96	<0.001	0.26	0.95
Chao1	895 ± 180	865 ± 178	731 ± 95.4	745 ± 174	777 ± 161	750 ± 192	639 ± 134	637 ± 67.1	<0.001	0.63	0.94
Shannon	8.99 ± 0.31	8.91 ± 0.28	8.69 ± 0.21	8.68 ± 0.35	8.72 ± 0.33	8.67 ± 0.35	8.53 ± 0.26	8.54 ± 0.13	<0.001	0.42	0.91
Pielou’s evenness	0.95 ± 0.01	0.94 ± 0.01	0.94 ± 0.01	0.94 ± 0.01	0.94 ± 0.01	0.94 ± 0.01	0.94 ± 0.01	0.94 ± 0.00	0.009	0.46	0.75
Simpson	0.997 ± 0.001	0.997 ± 0.001	0.996 ± 0.001	0.996 ± 0.001	0.997 ± 0.001	0.996 ± 0.001	0.996 ± 0.001	0.996 ± 0.000	0.002	0.39	0.78
Faith’s phylogenetic diversity	88.2 ± 12.9	84.9 ± 10.6	70.7 ± 6.33	73.7 ± 10.3	76.2 ± 7.79	77.0 ± 10.0	69.4 ± 8.35	65.8 ± 5.97	<0.001	0.92	0.29
Archaea											
Observed ASVs	45.7 ± 5.78	45.2 ± 6.64	41.0 ± 2.76	42.2 ± 7.47	48.0 ± 7.07	50.0 ± 6.86	40.4 ± 4.37	42.9 ± 4.27	<0.001	0.36	0.63
Observed genera	6.36 ± 1.36	6.00 ± 1.13	4.55 ± 0.82	5.33 ± 1.56	5.55 ± 1.13	5.58 ± 1.38	4.27 ± 1.19	3.67 ± 0.78	<0.001	0.85	0.06
Chao1	46.1 ± 5.9	45.4 ± 6.69	41.4 ± 3.05	43.7 ± 9.53	49.0 ± 7.78	51.6 ± 8.36	40.5 ± 4.48	43.1 ± 4.12	<0.001	0.39	0.65
Shannon	4.48 ± 0.18	4.57 ± 0.19	4.19 ± 0.32	4.43 ± 0.24	4.47 ± 0.25	4.66 ± 0.21	4.26 ± 0.20	4.41 ± 0.18	<0.001	0.003	0.45
Pielou’s evenness	0.81 ± 0.02	0.83 ± 0.02	0.78 ± 0.06	0.82 ± 0.03	0.80 ± 0.03	0.83 ± 0.03	0.80 ± 0.03	0.81 ± 0.03	0.10	<0.001	0.29
Simpson	0.93 ± 0.02	0.94 ± 0.01	0.90 ± 0.05	0.93 ± 0.01	0.91 ± 0.03	0.93 ± 0.02	0.90 ± 0.02	0.91 ± 0.02	<0.001	<0.001	0.31
Faith’s phylogenetic diversity	0.99 ± 0.13	0.92 ± 0.17	0.68 ± 0.11	0.75 ± 0.09	0.83 ± 0.15	0.87 ± 0.14	0.60 ± 0.18	0.58 ± 0.20	<0.001	0.91	0.42

^1^Throughout the annual cow-calf cycle, cows were subjected to four feeding regimens: drylot (DL), planted pasture (PP), summer native rangeland (SNR), and fall native rangeland (FNR)

^2^Residual feed intake adjusted for backfat thickness (RFIf) was measured during the drylot period. Cows were then classified into low RFIf (L) and high RFIf (H) groups based on their RFIf values

^3^*P*-values for overall differences among feeding regimens were obtained using the Friedman test, while differences between RFIf groups were assessed using the Wilcoxon rank-sum test. Interaction effects were evaluated by comparing generalized linear mixed models (GLMMs) with and without the interaction term using ANOVA

**Fig. 3:**
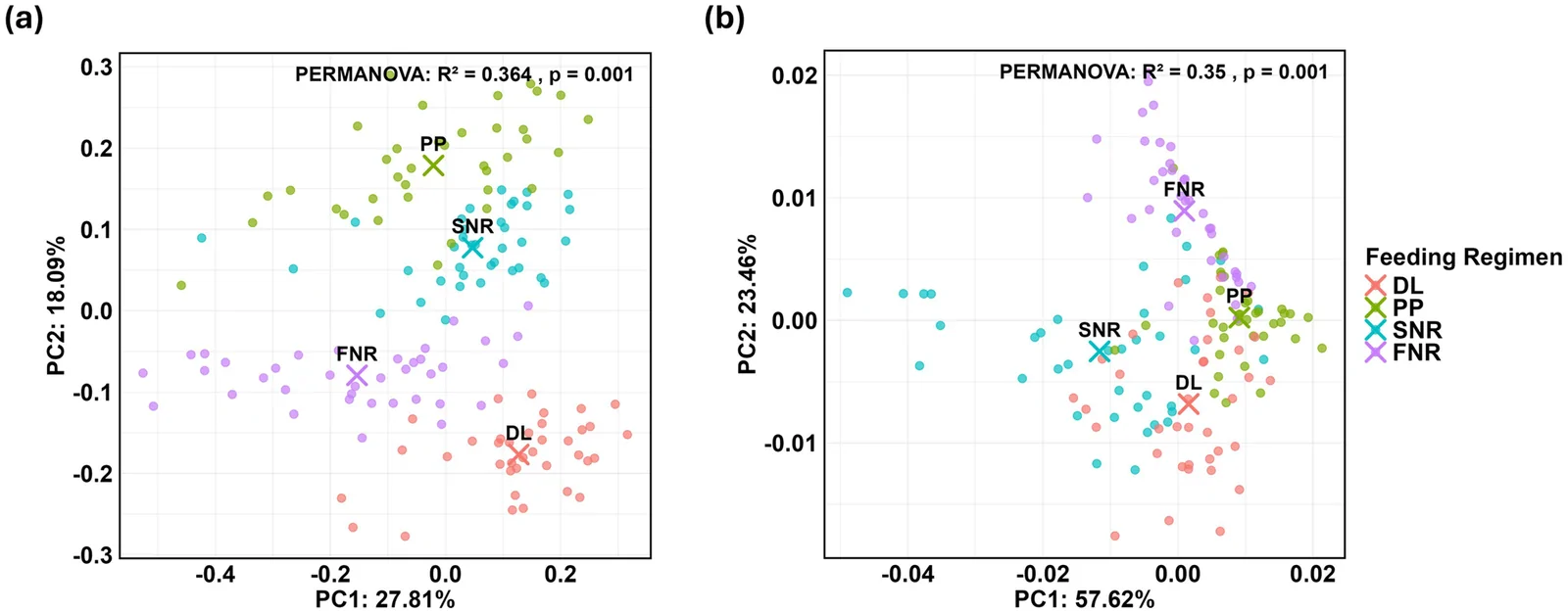
Beta diversities of rumen (**a**) bacterial and (**b**) archaeal communities across the feeding regimens. Weighted UniFrac distance were used for beta diversity analyses. Differences in beta diversity among feeding regimes were assessed using PERMANOVA. DL: drylot; PP: planted pasture; SNR: summer native rangeland; and FNR: fall native rangeland

Principal coordinate analysis with weighted UniFrac distances revealed that both bacterial and archaeal communities were separated across the feeding regimens (Fig. 3a, bacteria: R^2^ = 0.36, *p* < 0.001; Fig. 3b, archaea: R^2^ = 0.35, *p* < 0.001) but were not affected by RFIf (Fig. S1c and d; *p* > 0.10). Further pairwise-comparisons using PERMANOVA showed that the bacterial communities in the rumen of cows fed under DL were more unique than those under grazing regimes (R^2^ = DL vs. PP: 0.32, DL vs. SNR: 0.30, and DL vs. FNR: 0.33; *p* < 0.001). These differences were greater than the comparisons within the grazing periods (R^2^ = PP vs. SNR: 0.17, PP vs. FNR: 0.28, and SNR vs. FNR: 0.23; *p* < 0.001). In contrast, the archaeal communities in cows fed under DL were closer to those from the cows under PP and SNR but not FNR (R^2^ = DL vs. PP: 0.25, DL vs. SNR: 0.18, and DL vs. FNR: 0.38; *p* < 0.001). These differences were distinct from the comparisons within grazing periods (R^2^ = PP vs. SNR: 0.37, PP vs. FNR: 0.30, and SNR vs. FNR: 0.28; *p* < 0.001).

Based on SIMPER analysis, the taxa contributing most to the Bray–Curtis dissimilarity between residual feed intake groups were identified (Table S4). Bacterial differences were primarily associated with members of the *Absconditabacteriales* (SR1) lineage (average contribution = 0.038), the family *Prevotellaceae* (0.018), and the NK4A214 group within *Oscillospiraceae* (0.017), each showing modest contributions to the dissimilarity. Archaeal dissimilarity was mainly driven by two dominant *Methanobrevibacter* clades, *Methanobrevibacter gottschalkii* (0.040) and *Methanobrevibacter ruminantium* (0.034), which together accounted for 65.4% of the Bray–Curtis dissimilarity, with *Methanobrevibacter gottschalkii* contributing more in the high-RFIf group and *Methanobrevibacter ruminantium* in the low-RFIf group (Table S4).

### Microbial composition and temporal patterns across feeding regimens and RFIf groups

**Fig. 4:**
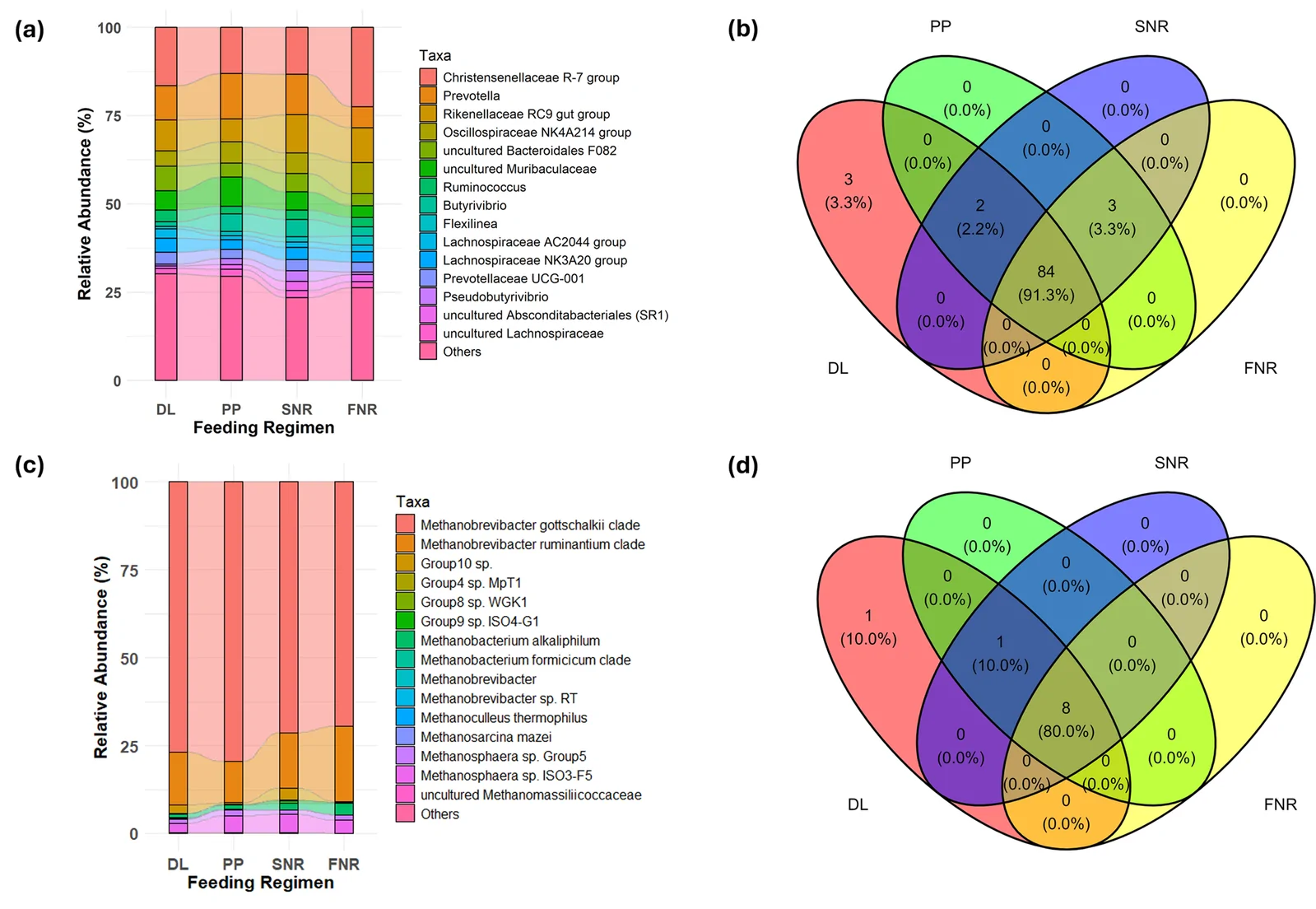
Taxonomic composition and shared taxa across the feeding regimens of the cow-calf production cycle: (**a**) relative abundances of bacterial taxa at genus level, (**b**) shared bacterial genera, (**c**) relative abundances of archaeal taxa at species level, and (**d**) shared archaeal species. In panels (**a**) and (**c**), less abundant taxa were consolidated into the ‘others’ category. DL: drylot; PP: planted pasture; SNR: summer native rangeland; and FNR: fall native rangeland

**Fig. 5:**
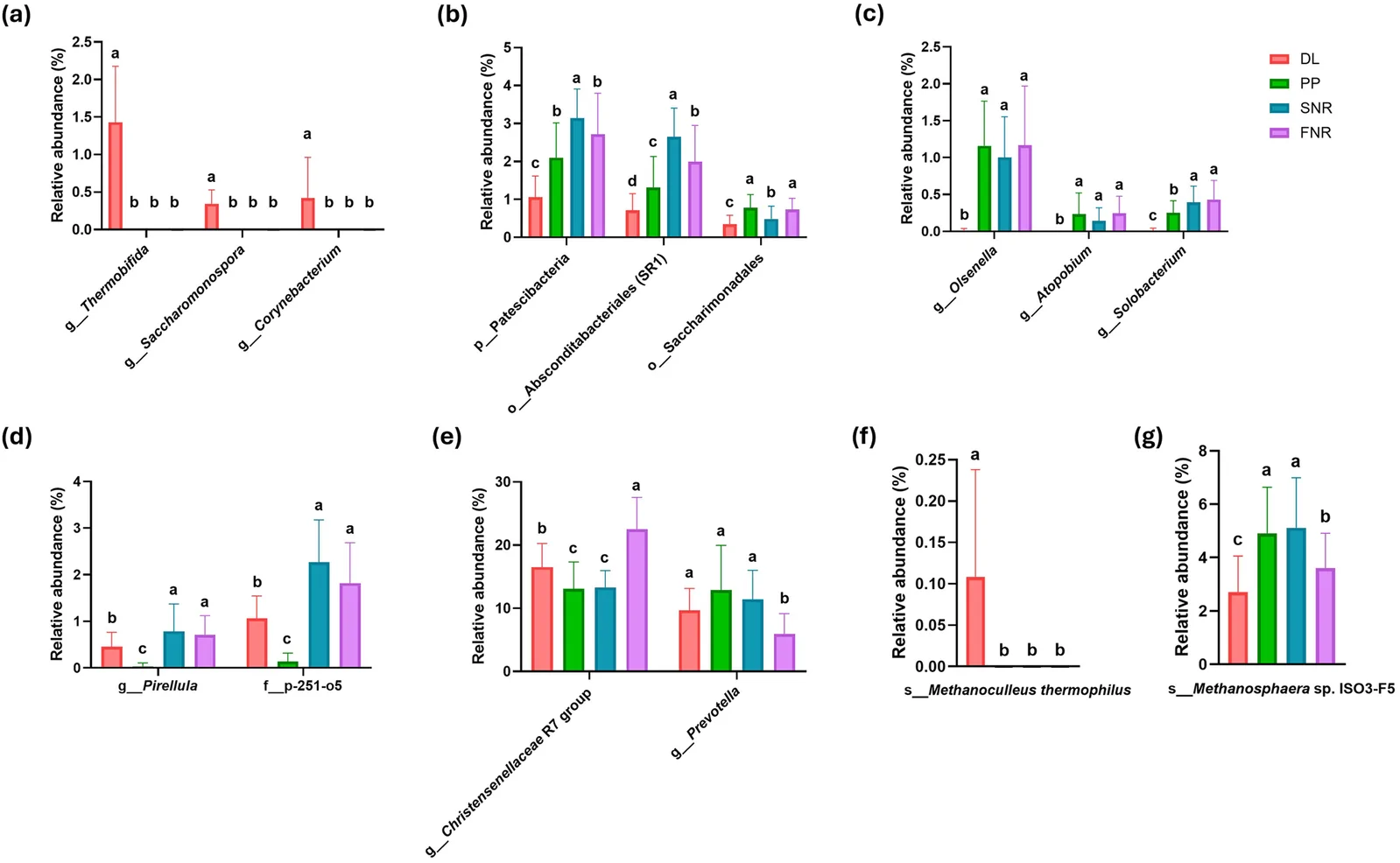
Differential abundance of bacterial and archaeal taxa between feeding regimens of the beef cow-calf production cycle: (**a**) DL-specific bacteria; (**b**) grazing abundant bacteria; (**c**) grazing-specific bacteria; (**d**) rumen heritable bacteria; (**e**) dominant bacteria; (**f**) DL-specific archaea; (**g**) RFIf-associated archaea. Different letters indicate significant differences (*Q* < 0.05) among the feeding regimens based on the linear mixed model analysis of Maaslin2. DL: drylot; PP: planted pasture; SNR: summer native rangeland; and FNR: fall native rangeland. Prefixes p_, o_, f_, g_, and s_ denote phylum, order, family, genus, and species, respectively

A total of 13 phyla and 92 genera were detected in the rumen bacterial communities of cows across the four feeding regimens. The most abundant phyla were Firmicutes (54.8%), Bacteroidota (38.2%), Patescibacteria (2.3%), Actinobacteriota (1.7%), Chloroflexi (1.5%), Planctomycetota (0.5%), and Proteobacteria (0.4%) across the feeding regimens. The 10 most abundant genera were *Christensenellaceae* R-7 group (16.4%), *Prevotella* (10.0%), *Rikenellaceae* RC9 gut group (9.0%), NK4A214 group (6.2%), uncultured *Muribaculaceae* genus (5.5%), F082 (4.9%), *Butyrivibrio* (3.4%), *Lachnospiraceae* NK3A20 group (3.3%), *Prevotellaceae* UCG-001 (3.0%), and *Ruminococcus* (2.7%) (Fig. 4a). Across all feeding regimens, 84 bacterial genera (91.3% of total genera) were detected in the rumen of cows. In the meantime, cows fed under DL had diet-specific genera (*Thermobifida*, *Saccharomonospora*, and *Corynebacterium*) that were not found later during grazing (Figs. 4b and 5a).

For rumen archaeal communities, one phylum (Euryarchaeota) and 10 species were found in cows across all four feeding regimens. *Methanobrevibacter gottschalkii* clade (74.3%), *Methanobrevibacter ruminantium* clade (16.0%), and *Methanosphaera* sp. ISO3–F5 (4.1%) were the most dominant species (Fig. 4c). Throughout all the feeding regimens, 8 archaeal species (80.0% of total species) were commonly detected, and *Methanoculleus thermophilus* was only found in the rumen of cows when fed under DL (Fig. 4d).

### Differential abundance of rumen bacterial taxa across feeding regimens

Differential abundance analysis (DAA) using MaAsLin2 revealed most rumen bacterial taxa (11 of 13 phyla and 90 of 92 genera) showed differential abundance across the feeding regimens (Table S5; *Q* < 0.10). Due to the large number of differentially abundant taxa identified across all feeding regimens, only a subset of differentially abundant bacteria is highlighted in Fig. 5. A total of 6 bacterial genera (*Colidextribacter*, FD2005, *Shuttleworthia*, *Oribacterium*, *Absconditabacteriales* (SR1), *Bacteroidales* BS11 gut group) and one phylum Actinobacteriota were consistently differentially abundant among all feeding regimens (Table S5; *Q* < 0.10).

Following the overall comparison, differential abundance of bacterial taxa between consecutive feeding regimens was examined to capture short-term microbial changes. From DL to PP, a total of 8 bacterial phyla and 76 genera and were identified as differentially abundant. Among them, the abundances of 1 phylum and 8 genera were changed as strongly (|coef| > 4), 2 phyla and 31 genera as moderately (2 ≤ |coef| < 4), and 5 phyla and 37 genera as weakly (|coef| < 2). Notably, genera *Colidextribacter*, FD2005, *Shuttleworthia*, and *Olsenella* showed significant increases in abundance (coef > 4, *Q* < 0.001), while genera *Papillibacter*, uncultured *Ruminococcaceae*, *p*-251-o5, *U29–B03* and phylum Proteobacteria were significantly decreased (coef < −4, *Q* < 0.001). *Thermobidifa* and *Saccharomonospora* exhibited decreases in abundance from DL to PP, with the most significant *Q*-values and moderate coefficient values (Fig. 5a; coef < −2, *Q* < 10^−45^).

In the comparison between PP and SNR, 7 phyla and 58 genera were identified as differentially abundant taxa. Among them, 1 phylum and 3 genera were classified as strongly, 9 genera as moderately, and 46 genera and 6 phyla as weakly differentially abundant taxa, respectively. Within the strongly and moderately differentially abundant taxa, the abundance of phylum Planctomycetota and genera *p*-251-o5, *Pirellula*, *Papillibacter* genera increased (coef > 3.5, *Q* < 0.001), whereas the abundances of 9 genera including *Colidextribacter*, *Shuttleworthia*, *Enterorhabdus*, *Ruminococcus gauvreauii* group, *Eubacterium nodatum* group, *Anaerorhabdus furcosa* group, *Defluviitaleaceae* UCG-011, Family XIII UCG-001, and uncultured *Erysipelatoclostridiaceae* exhibited significant decreases (coef < −2, *Q* < 0.001). Among them, *Pirellula* and *p*-251-o5 had the most significant *Q*-values (Fig. 5d).

Between SNR and FNR, a total of 8 phyla and 51 genera were identified as differentially abundant taxa. Only the genus FD2005 was classified as strongly differentially abundant taxon and showed a significant decrease in FNR (coef < −4, *Q* < 0.001). A total of 7 genera were classified as moderately differentially abundant, while 8 phyla and 43 genera were classified as weakly differentially abundant. Among the seven moderately differentially abundant genera, the relative abundance of *Defluviitaleaceae* UCG-011 and *Lachnospiraceae* UCG-006 increased (coef > 2, *Q* < 0.001), while those of *Oribacterium*, *Bacteroidales* BS11 gut group, *Pseudobutyrivibrio*, *Shuttleworthia*, and *Colidextribacter* showed significant decreases (coef < −2, *Q* < 0.001).

### Differential abundance of rumen archaeal taxa across feeding regimens

For rumen archaeal communities, all of the observed species except for *Methanosarcina mazei*, showed differential abundance across the feeding regimens (Table S6; *Q* < 0.10). Only Group9 sp. ISO4–G1, belonging to the family *Methanomassiliicoccaceae*, was consistently identified as differentially abundant among all feeding regimens (Table S6; *Q* < 0.10). From DL to PP, 8 species were identified as differentially abundant taxa. Among them, Group10 sp., belonging to the family *Methanomassiliicoccaceae*, was classified as a moderately differentially abundant taxon and its abundance significantly decreased (coef < −2, *Q* < 0.001) with other 7 species being classified as weakly differentially abundant taxa. Between PP and SNR, two species were classified as moderately and four as weakly of total six differentially abundant species. The abundances of both moderately differentially abundant species, Group10 sp. and Group9 sp. ISO4–G1, significantly increased (coef > 3, *Q* < 0.001) from PP to SNR. From SNR to FNR, seven species were differentially abundant. Among them, the abundance of Group10 sp. and Group9 sp. ISO4–G1 showed significant decrease which were classified as strongly and moderately (coef: −6.25 and coef: −2.75; *Q* < 0.001), while 5 species were classified as weakly differentially abundant.

### Differential abundance of rumen bacterial and archaeal taxa between different feed efficiency groups

In total, 2 bacterial genera and 1 archaeal species were found to be differentially abundant (*p* < 0.05and *Q* < 0.10) in the rumen of cows with different RFIf. The relative abundance of genus *Eubacterium cellulosolvens* group was significantly higher (coef: 0.56, *Q* = 0.028), and that of *Colidextribacter* tended to be higher in the rumen of H-RFIf cows than in the L-RFIf group (coef: 0.98, *Q* = 0.086). For archaeal communities, the relative abundance of *Methanosphaera* sp. ISO3–O5 was significantly higher (Fig. 5g; coef: 0.45, *Q* = 0.046) in the rumen of L-RFIf cows.

### Differential abundance of predicted rumen microbial functions between different feed efficiency groups

Functional profiles of the rumen microbiota were predicted using CowPI, which incorporates a rumen-specific database to improve accuracy in pathway inference (Table S7). In total, 8 predicted functional features were found to be significantly associated with RFIf (*p* < 0.05and *Q* < 0.10). Among KEGG modules, the relative abundance of Staphyloferrin B biosynthesis (M00875) was significantly lower (coef: −0.50, *Q* = 0.043), whereas CMP-Neu5Ac/Neu5Gc biosynthesis (M00955) and dTDP-Qui4NAc biosynthesis (M01026) were higher (coef: 0.84, *Q* = 0.060; coef: 0.02, *Q* = 0.074) in L-RFIf cows. At the KEGG pathway level, cutin, suberin and wax biosynthesis (ko00073) was significantly lower in L-RFIf cows (coef: −0.62, *Q* = 0.026). From the CowPI predictions, the p53 signaling pathway was significantly lower in L-RFIf cows (coef: −0.62, *Q* = 0.016). The abundance of glycosaminoglycan biosynthesis – chondroitin sulfate was also lower (coef: −0.047, *Q* = 0.050). Similarly, polycyclic aromatic hydrocarbon degradation (coef: −0.35, *Q* = 0.074) and other ion-coupled transporters (coef: −0.015, *Q* = 0.074) were lower in L-RFIf cows.

### Relationships between VFA concentrations and rumen prokaryotic diversity and taxa

Spearman’s rank correlation analysis revealed that bacterial Chao1, observed ASVs, Faith’s PD, and archaeal Faith’s PD and Simpson indices had moderate positive correlations with ruminal iso-butyrate and iso-valerate concentrations (ρ > 0.3, *p* < 0.001), while archaeal Chao1, observed ASVs, and Faith’s PD indices had moderate negative correlations with ruminal propionate proportion (ρ < −0.3, *p* < 0.0001) (Table S8). Further analysis showed 13 bacterial genera showed moderate positive correlations, and 9 genera showed moderate negative correlations with total VFA concentration (Table S9). Among them, the Prevotellaceae YAB2003 group (ρ = 0.56, *Q* < 0.0001) and *Prevotella* (ρ = 0.52, *Q* < 0.0001) showed the strongest positive correlations, while others exhibited weak correlations (ρ < 0.5) with total VFA concentration. For archaea, Group 4 sp. MpT1 showed a moderate positive correlation (ρ = 0.50, *Q* < 0.0001), while *Methanobrevibacter ruminantium* clade exhibited a moderate negative correlation (ρ = −0.30, *Q* < 0.0001) with total VFA concentration. Additionally, 6 bacterial genera showed moderate positive correlations, and 6 genera showed moderate negative correlations with concentrations of all individual VFAs (acetate, propionate, butyrate, iso-butyrate, valerate, iso-valerate). Among them, *Prevotella* (ρ > 0.49, *Q* < 0.0001) and *Bacteroidales* RF16 group (ρ > 0.46, *Q* < 0.0001) had the strongest positive correlations (ρ > 0.45) with all individual VFAs, while *Christensenellaceae* R-7 group (ρ < −0.48, *Q* < 0.0001) had strong negative correlations (ρ < −0.45) with all individual VFAs. The archaeal taxa Group 4 sp. MpT1 had strong positive correlations (ρ > 0.46, *Q* < 0.0001) with all individual VFAs.

## Discussion

This study assessed the rumen microbiome of beef cows across different feeding regimens of the year-round cattle production cycle. Grazing is a common cattle production practice that allows domesticated herbivores to selectively feed on various habitat types and plant species. Specific grazing practices for beef cattle vary widely, depending on the availability and quality of forage, which are influenced by phenological development across the growing season, environmental attributes such as forage composition and soils, as well as management practices [48–50]. In our study, the seasonal changes in feeding regimens when transitioning from DL in winter, through PP in spring, to SNR in summer and FNR in fall, involve not only variation in feed types, forage quality, and animal physiology (pregnancy and calving of cows) but also changes in environmental factors, such as animal density and water source.

A limitation of this study is that the detailed nutritional composition of forages (e.g., NDF, ADF, and CP) during PP, SNR, and FNR was not directly measured, while such data were available for the DL period. The complex and diverse plant communities across native rangelands, combined with the selective grazing behavior of cattle, make systematic forage nutritional profiling challenging in free-range grazing systems. However, based on the plant species observed on pasture and their growth cycle, it was expected that plants on PP contain higher protein and less fibre; plants on SNR contain higher structural fibre (increased cellulose/NDF/ADF); whereas plants on FNR are mature or dormant and contain even higher fibrous compounds (e.g. cellulose and lignin). Consequently, we hypothesize that the digestibility of the consumed plants decreases from PP to SNR to FNR, although they may all remain higher than that of the DL diet. Future studies should include forage sampling and nutritional analysis across grazing periods to more directly link forage quality to rumen microbial shifts.

The identified major bacterial phyla Firmicutes (54.8 ± 9.19%) and Bacteroidetes (38.2 ± 10.0%), and the methanogen genus *Methanobrevibacter* (90.3 ± 3.16%) are consistent with the dominant rumen bacterial and archaeal taxa reported in other beef and dairy cattle studies [51–56]. A notable characteristic of the rumen microbiota in grazing beef cows was the high proportion of the genus *Christensenellaceae* R-7 group (16.4 ± 5.48%), and the abundance of this taxon increased (coef: 0.75, *Q* < 0.0001) when transitioning from SNR to FNR (Fig. 5e). The *Christensenellaceae* R-7 group is an uncultivated genus belonging to the phylum Firmicutes and has been consistently detected in the rumen not only of beef and dairy cattle but also of other ruminants such as yaks, buffalo, and sheep [57–61]. Notably, it has been shown to be more abundant in the rumen of ruminants fed fibre-rich diets than in those of animals fed concentrate-rich diets, suggesting its potential role in fibre degradation [61–63]. Research on yaks raised in native grazing environments has consistently reported the presence of the *Christensenellaceae* R-7 group in the rumen of yaks [57, 64–67]. For example, Hu et al. [68] reported that the *Christensenellaceae* R-7 group was more abundant in the rumen of yaks than in feedlot beef cattle, and was especially enriched in yaks grazing on native rangelands compared to yaks fattened in feedlots (*p* = 0.018). As the accessible feed for wild animals usually contains more low-quality fibre, these findings suggest that the *Christensenellaceae* R-7 group may have developed better capacity to utilize structural fibrous compound compared to other bacteria. This is further supported by the positive correlation observed between *Christensenellaceae* R-7 group and molar proportion of acetate (ρ = 0.52, *Q* < 0.001), consistent with its role in fibre-associated fermentation under grazing conditions. Thus, despite genetic and environmental differences between yaks and beef cows, this bacterial group is expected to have a significant function in ruminants grazing in dormant native rangelands.

Bacterial genera *Thermobifida*, *Saccharomonospora*, and *Corynebacterium* were exclusively found in the rumen of cows fed under DL (Fig. 5a). Although these genera have previously been detected in the rumen [69, 70], it remains unclear whether they act merely as passengers or actively contribute to fibre degradation or other functions in the rumen. These genera, which belong to the phylum Actinobacteriota, possess lignocellulosic biomass-degrading capabilities and can form spores, enabling them to survive in harsh environments [69, 71–74]. These characteristics suggest that these taxa may play a role in lignocellulose degradation in the rumen of grazing cows. Similarly, *Methanoculleus thermophilus* was found exclusively in DL (Fig. 5f), which was identified as dominant methanogens in the rumen of ruminants raised under intensive livestock farming conditions, such as high-density confined feeding operations, in Asian countries including Korea and Japan [75–77]. *Thermobifida* and *Saccharomonospora* were negatively correlated with propionate and butyrate, but strongly positively correlated with iso-butyrate and iso-valerate. As iso-acids indicate branched-chain amino acid fermentation, these taxa may reflect fermentation environments associated with silage-based DL feeding, where ensiling processes release di- and oligo-peptides from forage [78], rather than lignocellulose degradation. In contrast, the ecological role of *Methanoculleus thermophilus* in the rumen remains unclear. Further studies are needed to fully understand whether these prokaryotic taxa are environment-derived organisms and to clarify their specific metabolic roles in the rumen through functional approaches such as isolation, cultivation, or metagenomic analyses.

Throughout the year-long production cycle, the phylum Patescibacteria was more abundant (*Q* < 0.0001) in the rumen of cows under grazing periods (PP, SNR, and FNR) compared to DL (Fig. 5b). This phylum has been detected in the rumen of various ruminants, including beef and dairy cattle and yak, with reported proportions ranging from 0.03% to 16% of the total rumen bacterial community [79–84]. For instance, Zhang et al. [85] observed that the abundance of Patescibacteria peaked in yaks during grazing and declined gradually over a 16-day feedlot period. Although the ecological roles of Patescibacteria in the rumen remain unclear, certain species within this phylum exhibit auxotrophic features, relying on other microorganisms for essential biosynthetic functions. These species have been identified in various environments, including freshwater, soil, the human oral cavity, and the gut of mammals [86, 87]. The auxotrophic characteristics of this phylum may enable symbiotic or parasitic interactions with polysaccharide-degrading bacteria. This relationship could potentially reduce host FE, as these organisms utilize intermediates or end-products of the polysaccharide degradation as their energy sources [82, 88]. Supporting this, Ma et al. [64] reported significantly higher Patescibacteria abundance (*p* < 0.05) in growth-retarded yaks compared to normal yaks. Although the study of Patescibacteria is challenging due to their resistance to cultivation and dependence on host bacteria, future research is needed to explore Patescibacteria in different cattle production environments and examine their impact on the rumen microbiome and the performance of cattle while grazing.

Similarly, the genus *Olsenella* also showed significantly higher abundance (*Q* < 0.0001) during grazing periods (PP, SNR, and FNR) compared to DL (Fig. 5c). *Olsenella*, a member of the family *Atopobiaceae* within the phylum Actinobacteriota, is a moderately obligate anaerobic bacterium commonly found in the gastrointestinal tracts of homeothermic vertebrates, including the rumen of ruminants. Additionally, *Olsenella* has been identified as one of the primary colonizing microbes on plant cell walls in the rumen. It plays a dominant role in the initial fibre breakdown process (0–8 hr after feeding), with peak abundance during the primary colonization phase (2 hr after feeding) [89]. These findings suggest that *Olsenella* possesses the capacity to degrade and utilize the surface structures of plant fibres, including hemicellulose, proteins, and lipopolysaccharides, during the initial fibre breakdown process. Furthermore, *Olsenella*’s peptidolytic and mucin-utilizing capabilities [90], as well as its presence in rumen epithelial-adherent (epimural) communities alongside genus *Atopobium* [91, 92], suggest that members of the family *Atopobiaceae* could facilitate attachment to, and degradation of, host glycoproteins, as well as plant cell wall surfaces and structural proteins. The significant increases of *Atopobium* (coef: 2.88, *Q* < 0.0001), *Olsenella* (coef: 4.31, *Q* < 0.0001), and *Solobacterium* (coef: 3.57, *Q* < 0.0001) in PP compared to DL (Fig. 5c), which undergoes considerable losses of hemicellulose and other soluble proteins during the ensiling process from silage microbiota [93]. Furthermore, these findings highlight potential interactions between rumen planktonic and epimural communities, warranting further investigation into their ecological relationships and contributions to rumen function of grazing cows.

Native rangeland (SNR and FNR) differs markedly from planted pasture (PP) in vegetation composition, diversity and seasonal variations in forage quality. The increase in the abundance of woody forages (shrubs and trees) coupled with advancing senescence with phenological changes, are all potentially factors that increase plant fibre contents, including that of less digestible carbohydrates. Decreased rumen bacterial populations in SNR and FNR (Fig. 2) indicate a more stressful environment, likely due to increased structural carbohydrates in the ingested feed, the reduction of high-quality forage availability caused by advanced plant senescence (especially in FNR), or the presence of secondary metabolites in native plants. Under these conditions, *Pirellula* (coef: 4.24 and 4.16, *Q* < 0.0001) and *p*-251-o5 group (coef: 5.43 and 5.03, *Q* < 0.0001) exhibited significantly higher abundance compared to PP (Fig. 5d), when the latter would have coincided with the most rapid period of pasture growth and particularly high forage quality levels [94, 94]. *Pirellula*, a member of the phylum Planctomycetota, predominantly inhabits carbon-rich environments [95]. It has been identified in rumen microbiomes across various ruminants [96–99], although its specific functions remain undetermined. In dairy cows, *Pirellula* exhibits high heritability (h^2^ > 0.5) and shows a strong correlation with *p*-251-o5, the most heritable taxon (h^2^ = 0.79) [97, 100]. Previous research showed that *p*-251-o5 was negatively correlated with VFA concentrations, particularly propionate and valerate [100]. Furthermore, Mu et al. [101] observed that during high-grain diet-induced acidosis, *p*-251-o5 abundance significantly decreased (*p* < 0.001) as propionate and valerate concentrations increased. Consistent with these findings, our study revealed negative correlations (ρ < −0.3, *Q* < 0.001) between *p*-251-o5/*Pirellula* and all VFAs (Table S9). These correlations, along with the increased abundance of these taxa in native rangelands with higher fibre content of forages, especially in FNR, suggest that these heritable taxa are associated with cows consuming diets rich in structural carbohydrates. This is in contrast to diets rich in soluble carbohydrates, such as those in PP, while their specific metabolic roles require further investigation.

The shift in foraging from SNR to FNR might involve a reduction in forage quality due to plant senescence, leading to higher lignocellulose content, which could significantly affect the rumen microbiome. During this transition, *Christensenellaceae* R-7 group increased substantially (13.3% to 22.5%; coef: 0.75, *Q* < 0.0001), while *Prevotella* decreased significantly (11.4% to 5.9%; coef: −1.01, *Q* < 0.0001). Both bacterial taxa *Christensenellaceae* and *Prevotella* have been noted as gut microbiome components associated with host health [102, 103]. In the rumen, *Christensenellaceae* has consistently been reported as one of the dominant microbes, often alongside *Prevotella* and *Rikenellaceae* RC9 gut group [63, 65, 104, 105]. In both beef and dairy cattle, *Christensenellaceae* exhibits heritable traits, and shows a positive correlation with acetate and a negative correlation with propionate [97, 106]. Several studies have demonstrated that ruminal abundance of the *Christensenellaceae* R-7 group increases with higher forage:concentrate ratios [63, 67, 101]. Bach et al. [107] tracked microbiome changes in dairy cows during the peri-parturient period and observed a significant decrease in *Christensenellaceae* R-7 group after calving (15.1% to 4.2%; *p* < 0.00001), which coincided with a dietary shift from a high-NDF diet (NDF: 51.2%) to a low-NDF diet (NDF: 34.8%). These results align with our findings that the *Christensenellaceae* R-7 group abundance decreased (coef: −0.38, *Q* < 0.0001) when transitioning from DL to PP, with negative correlations (ρ < −0.45, *Q* < 0.0001) to all VFAs (Table S9). Unlike the *Christensenellaceae* R-7 group, *Prevotella* has shown variable responses to changes in forage:concentrate ratios in previous studies. This variability may stem from *Prevotella'*s resistance to low pH and extensive functional redundancy, enabling it to utilize diverse plant polysaccharides [108, 109]. The inconsistent responses of dominant rumen microbes to various feeding regimens might reflect their distinct niche specifications, as *Christensenellaceae* R-7 group specializes in lignocellulose degradation and *Prevotella* specializes in hemicellulose utilization [67, 110]. Future research exploring the relationship between specific niches of dominant rumen microbes and feed sources in DL and grazing environments could provide valuable insights into how diverse native rangelands affect the nutrition of grazing cows, while also highlighting the potential value of native rangelands as a sustainable feed source.

On top of examining the rumen microbiota shifts among feeding regimes, the current study also compared the rumen microbial responses to such changes in animals belonging to different feed efficiency group. It is noticeable that RFIf was only measured when cows were under DL condition but not on pasture, therefore the grouping of the animals to H-RFIf and L-RFIf primary reflected cattle feed efficiency prior to the grazing period. However, as reported in previous studies, RFI measured under confined conditions was positively correlated with RFI under grazing conditions or that relative intake differences were maintained [111, 112]. Based on this, major reranking of RFI between DL, PP, SNR, and FNR systems in the current study were expected to be limited, and as such, the comparison between H-RFIf and L-RFIf was still of value to understand the microbial adaption in animals bred for different feed efficiency.

Of the identified rumen microbes, higher abundance of the *Eubacterium cellulosolvens* group and *Colidextribacter* were observed in the rumen of H-RFIf cows. *E. cellulosolvens*, a predominant cellulolytic bacterium in the rumen, is known for its acidic endoglucanase activity. Unlike other cellulolytic bacteria that lose activity at low pH, *E. cellulosolvens* exhibits peak activity at pH 4, attributed to its acidophilic enzyme rCel5A [113, 114]. This acidophilic activity aligns with the increased abundance of *E. cellulosolvens* observed in PP. Increased feed intake after calving and abundant soluble nutrients in the vegetative stage of planted grasses in PP might contribute to rapid fermentation and higher VFA production, which are followed by transient low pH conditions [94, 115]. In addition, *E*. *cellulosolvens* has been reported to produce lactate and butyrate as major fermentation products, which is consistent with its positive correlation with butyrate in this study (ρ = 0.54, Q < 0.001) [19]. In contrast to *Eubacterium*, *Colidextribacter* remains largely unexplored in the rumen. It has been associated with high-grain diets, exhibiting a rapid digestion response with peak abundance within 12 hr of feeding [116, 117]. This suggests that *Colidextribacter* may function as an amylolytic bacterium. Notably, this is consistent with its negative correlation with molar proportion of acetate (ρ = −0.45, *Q* < 0.001) and A:P ratio (ρ = −0.24, *Q* = 0.009) and positive correlation with molar proportion of butyrate (ρ = 0.56, *Q* < 0.001), a VFA pattern typically associated with non-structural carbohydrate fermentation [117]. Given their small proportional abundance in the rumen microbiome (0–2%), these bacteria may not directly influence FE. Instead, changes in their abundance might be an outcome of the differences in feeding behavior between RFIf groups, particularly in PP [118]. Comprehensive studies examining interactions among feeding behavior, rumen microbiota, microbe-derived products (e.g., VFAs, methane, and lipopolysaccharide), and host physiology, are needed to elucidate their roles in FE.

**Fig. 6:**
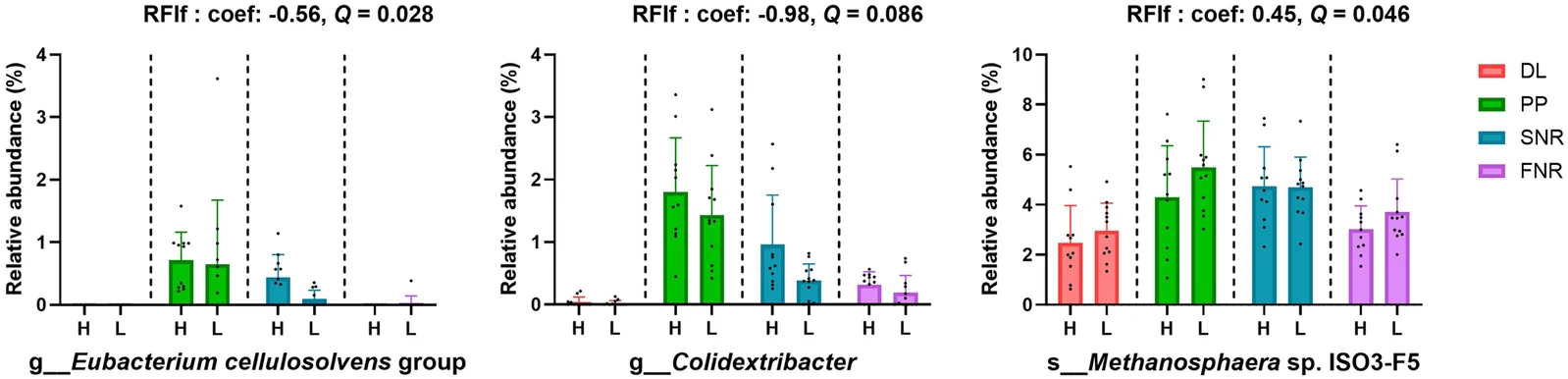
The changes in differential abundant bacterial and archaeal taxa between high RFIf (H-RFIf) and low RFIf (L-RFIf) groups. The significant values (*Q*) are from linear mixed model analysis of Maaslin2. Prefixes g_ and s_ denote genus and species, respectively

In contrast to the high diversity observed in rumen bacterial communities, rumen archaea are predominantly represented by two major clades: *Methanobrevibacter gottschalkii* clade (74.3 ± 6.9%) and *Methanobrevibacter ruminantium* clade (16.0 ± 5.8%), consistently reported as dominant rumen methanogens across various studies. Additionally, methylotrophic archaea such as *Methanosphaera* and the family *Methanomassiliicoccaceae*, formerly known as the rumen cluster C group, have also been observed alongside the Methanobacteriales, the order containing *Methanobrevibacter* [75, 119–121]. The DAA in our study revealed that *Methanosphaera* sp. ISO3–F5 was more abundant in the rumen of L-RFIf cows than in that of H-RFIf cows (Fig. 6; coef: 0.45, *Q* = 0.046), and showed increased abundance in PP and SNR compared to DL and FNR (Fig. 5g). Previous studies on feedlot steers show inconsistent relationships between RFI and *Methanosphaera stadtmanae* [23, 122]. *Methanosphaera* is a methylotrophic methanogen that utilizes methanol [120, 123]. Recent studies revealed *Methanosphaera* sp. ISO3–F5 is a methanol-dependent methanogen [124, 125] and suggested its potential relationship with pectin degrading bacteria including *Prevotella* and *Butyrivibrio* which provide methanol from pectin by their pectin methyl esterases [126]. In the current study, the decreased abundance of *Methanosphaera* sp. ISO3–F5 and the pectin-degrading bacteria *Prevotella* and *Butyrivibrio* in FNR compared to PP (coef < −0.8, *Q* < 0.01), may be attributed to forage maturation. This maturation increases lignin content and thereby hinders hemicellulose and pectin degradation [127]. Although a direct link among *Methanosphaera* sp. ISO3–F5, ruminal methanol metabolism, and host feed efficiency has not been established, we hypothesize that this methanol-dependent methanogen may indirectly contribute to the degradation of plant cell wall components, including pectin, by removing methanol as a by-product of pectin fermentation. Future studies are needed to verify this potential ecological role through functional analyses.

It is interesting that the DA patterns of each of these microbes were similar across feeding regimes (Fig. 6), suggesting their resilience to environmental/dietary changes. As measuring RFIf during grazing were not feasible with our farm settings, it may be worth to measure the body weight gain of the animals during grazing and use it as a proxy of feed efficiency of the animals in future studies. It may also be useful to evaluate whether these DA microbes identified in the current study also influence the body weight gain of the animals. If their roles in influencing host feed efficiency can be verified, they may then serve as the microbial markers for breeding. Additionally, eukaryotic microbes play important roles in digesting fibres. Exploring the composition and functions of the eukaryotic microbiota, as well as their interaction with prokaryotic microbiota, may further provide a more complete understanding of the comprehensive mechanisms of rumen microbiome under grazing.

## Conclusion

This study revealed several key microbial responses in the rumen of cows through a year-round production cycle, highlighting the variation in rumen microbiome composition of beef cows across different seasonal feeding regimens in a typical beef cow-calf production cycle in temperate rangelands of Western Canada. Furthermore, we identified taxa associated with RFIf defined under DL, whose DA pattern remained similar across feeding regimens, warrants further investigation on their impact on host feed efficiency. As the first comprehensive characterization of the rumen microbiome of beef cows under confined and grazing conditions in temperate climates, we found that grazing systems exhibited three distinctive microbial features, including enrichment of fibre-degrading taxa (*Christensenellaceae* R-7 group), increased abundance of taxa potentially processing plant secondary metabolites (Patescibacteria), and functional expression of highly heritable taxa (*p*-251-o5, *Pirellula*) under native rangeland conditions. Our findings offer a framework for understanding microbiome dynamics in cow-calf operations. Moreover, understanding which efficiency-associated microbes persist or change across grazing and confinement periods may support future development of microbiome-based strategies, such as targeted probiotics or diet formulations, to maintain beneficial microbial communities throughout the production cycle. Despite limitations such as the lack of detailed diet composition data and the absence of RFI measured in grazing environments, this study highlights the importance of longitudinal, landscape-level analyses of the rumen microbiome for grazing ruminants. Future integration of metagenomics and metabolomics at the genome and pathway level is needed to identify the functional capacities of these taxa, determine whether they are involved in fibre degradation and fermentation, and clarify how these microbial functions are linked to feed efficiency under different feeding regimens, ultimately informing sustainable beef production strategies.

## Electronic supplementary material

Below is the link to the electronic supplementary material.

Supplementary Material 1

Supplementary Material 2

Supplementary Material 3

## Data Availability

Raw amplicon sequencing data of rumen prokaryotes (bacteria and archaea) have been deposited at the National Center for Biotechnology Information (NCBI) under project number (PRJNA1248015).
