# Supplementary material for: Shifts in rumen prokaryotic communities of grazing beef cows with divergent feed efficiency in response to seasonal grazing
Source: Anim Microbiome. 2026 Nov 10;8:118. doi: 10.1186/s42523-026-00581-6 (PMC13753285; doi:10.1186/s42523-026-00581-6)
Supplement: Supplementary file 1 [file 42523_2026_581_MOESM1_ESM.pdf]

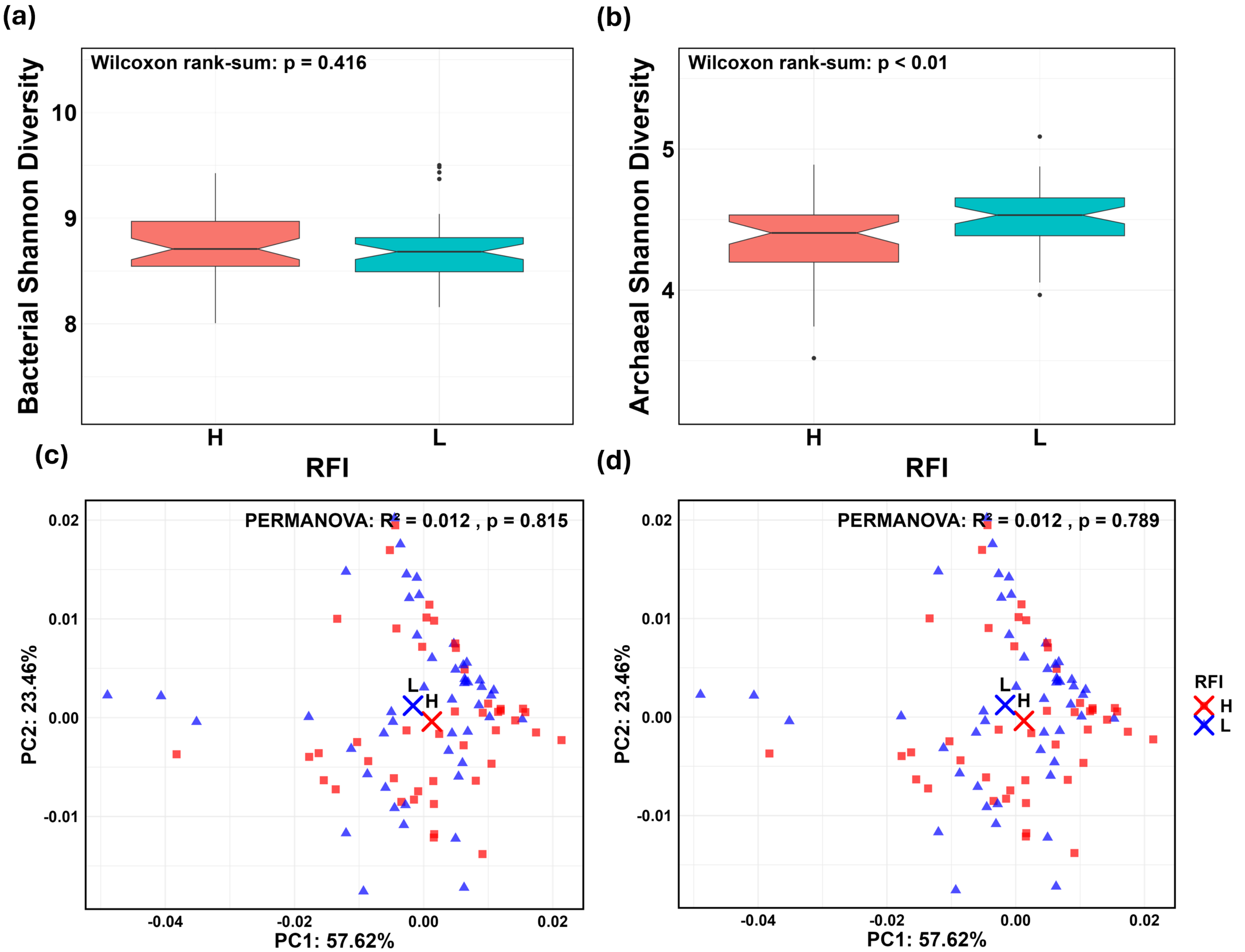

(a)
Wilcoxon rank-sum: p = 0.416
Bacterial Shannon Diversity
10
9
8
H
L
RFI
(b)
Wilcoxon rank-sum: p < 0.01
Archaeal Shannon Diversity
5
4
H
L
RFI
(c)
PERMANOVA: R² = 0.012 , p = 0.815
PC2: 23.46%
PC1: 57.62%
0.02
0.01
0.00
-0.01
-0.04
-0.02
0.00
0.02
L
H
(d)
PERMANOVA: R² = 0.012 , p = 0.789
PC2: 23.46%
PC1: 57.62%
0.02
0.01
0.00
-0.01
-0.04
-0.02
0.00
0.02
L
H
RFI
H
L
