# Supplementary material for: Shifts in rumen prokaryotic communities of grazing beef cows with divergent feed efficiency in response to seasonal grazing
Source: Anim Microbiome. 2026 Nov 10;8:118. doi: 10.1186/s42523-026-00581-6 (PMC13753285; doi:10.1186/s42523-026-00581-6)
Supplement: Supplementary file 2 [file 42523_2026_581_MOESM3_ESM.docx]

**Supplementary Tables**

**Table S1.** RFIf values of all animals and the RFIf classification of the selected animals.

**Table S2.** Denoising stats of bacterial 16S rRNA gene amplicon sequencing by DADA2.

**Table S3.** Denoising stats of archaeal 16S rRNA gene amplicon sequencing by DADA2.

**Table S4.** Similarity percentage analysis of taxa contributing to Bray–Curtis dissimilarity between residual feed intake groups in bacterial and archaeal communities.

**Table S5.** Differential rumen bacterial taxa across the grazing seasons.

**Table S6.** Differential rumen archaeal taxa across the grazing seasons.

**Table S7**. The changes in differentially abundant predicted functions between high RFIf (H-RFIf) and low RFIf (L-RFIf) groups inferred by PICRUSt2 and CowPI. The significant values (Q) are from linear mixed model analysis of Maaslin2.

**Table S8.** Correlation results between VFA profile and prokaryotic alpha diversity.

**Table S9.** Correlation results between VFA profile and prokaryotic taxa.

**Supplementary Figures**

**Figure S1.** Alpha and beta diversity comparisons of rumen microbial communities between high RFIf (H-RFIf) and low RFIf (L-RFIf) groups: (a) bacterial alpha diversity, (b) archaeal alpha diversity, (c) bacterial beta diversity, and (d) archaeal beta diversity of rumen communities. Shannon index was used for alpha diversity analyses and weighted UniFrac distances for beta diversity analyses. Statistical comparisons were performed using Friedman test for alpha diversity and PERMANOVA for beta diversity.
